# Mononuclear and Dinuclear Manganese(II) Complexes from the Use of Methyl(2-pyridyl)ketone Oxime

**DOI:** 10.1155/2010/960571

**Published:** 2010-07-04

**Authors:** Constantinos G. Efthymiou, Vassilios Nastopoulos, Catherine Raptopoulou, Anastasios Tasiopoulos, Spyros P. Perlepes, Constantina Papatriantafyllopoulou

**Affiliations:** ^1^Department of Chemistry, University of Patras, 265 04 Patras, Greece; ^2^Department of Chemistry, University of Florida, Gainesville, FL 32611-7200, USA; ^3^Institute of Materials Science, National Centre of Scientific Research “Demokritos”, 153 10 Aghia Paraskevi Attikis, Greece; ^4^Department of Chemistry, University of Cyprus, 1678 Nicosia, Cyprus

## Abstract

The reactions of methyl(2-pyridyl)ketone oxime, (py)C(Me)NOH, with manganese(II) sulfate monohydrate have been investigated. The reaction between equimolar quantities of MnSO_4_ · H_2_O and (py)C(Me)NOH in H_2_O lead to the dinuclear complex [Mn_2_(SO_4_)_2_{(py)C(Me)NOH}_4_] · (py)C(Me)NOH, **1** · (py)C(Me)NOH, while employment of NaOMe as base affords the compound [Mn(HCO_2_)_2_{(py)C(Me)NOH}_2_] (**2**). The structures of both compounds have been determined by single crystal X-ray diffraction. In both complexes, the organic ligand chelates through its nitrogen atoms. The IR data are discussed in terms of the nature of bonding and the structures of the two complexes.

## 1. Introduction

There is currently a renewed interest in the coordination chemistry of oximes [1]. The research efforts are driven by a number of considerations. One of these is that they are considered to be reasonable models for the biologically significant imidazole donor group of the amino acid histidine [2]. Thus, they potentially can be used for the synthesis of various nuclearity metal clusters to model M_x_ sites in biomolecules, including elucidating the structure and mechanism of action of the CaMn_4_ core of the water oxidizing complex within the photosynthetic apparatus of green plants and cyanobacteria [3, 4]. In addition, metal complexes of oximes can be used in several other applications, that is, the solution of pure chemical problems [5, 6], the development of new oxygen activation catalysts based on nickel(II) polyoximate complexes [7] and the application of metal ion/oxime systems as efficient catalysts for the hydrolysis of organonitriles [8]. In the latter, metal ions can behave as extremely strong activators of RCN molecules towards nucleophilic attack by OH^−^/H_2_O. Other applications of metal complexes of oximes include the design of Ca^2+^- and Ba^2+^-selective receptors based on site-selective transmetallation of polynuclear zinc (II)/polyoxime complexes [9], the study of metal-ion assisted organic transformations [10], and the mechanistic investigation of corrosion inhibition by Acorga P5000 (a modern corrosion inhibitor comprising 5-nonylsalicylaldoxime as a mixture of carbon-chain isomers) on iron surfaces [11]. Note also that oximate ligands are employed in the synthesis of homo- and heterometallic [1, 12] clusters and coordination polymers [13] with interesting magnetic properties, including single-molecule magnetism [14–16], and single-chain magnetism [17] behavior. 

**Scheme 1:**
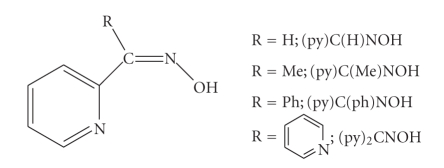
General structural formula and abbreviations of simple 2-pyridyl oximes, including methyl(2-pyridyl)ketone oxime [(py)C(Me)NOH].

Ligands containing one oxime group and one pyridyl group, without other donor sites, are popular in coordination chemistry. Metal-free pyridine oximes exhibit a plethora of biological properties including action on the cardiovascular system, sedative, antidepressant, antispasmodic, cytotoxic, antiviral, and bactericidal activities, while they are good antidotes for poisoning by organophosphorus compounds [18]. Most of these ligands contain a 2-pyridyl group, and thus are named 2-pyridyl oximes, (py)C(R)NOH (Scheme 1). The anionic forms of these molecules, (py)C(R)NO^−^, are versatile ligands for a variety of research objectives, including *μ*_2_ and *μ*_3_ behaviour; the activation of 2-pyridyl oximes by 3d-metal centers towards further reactions is also becoming a fruitful area of research. The majority of the metal complexes of these ligands have been prepared in the last 15 years and much of their chemistry remains to be explored in more detail [1].

**Scheme 2:**
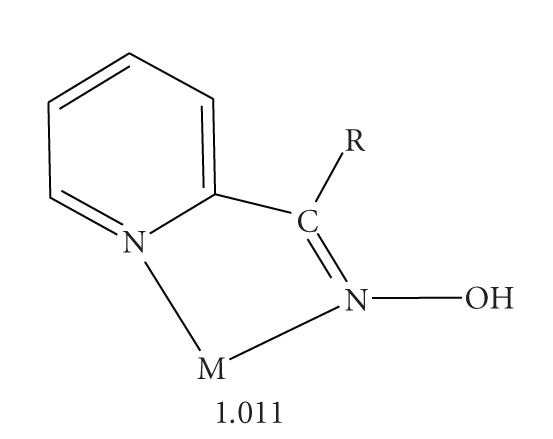
The common coordination mode of the neutral 2-pyridyl oximes and the Harris notation [26] which describes this mode.

With only few exceptions [19, 20], the hitherto structurally characterized metal complexes containing *neutral* 2-pyridyl oximes as ligands are *mononuclear*. The donor atoms of the neutral 2-pyridyl oximes in metal complexes are the nitrogen atom of the oxime group and the nitrogen atom of the pyridyl group. Thus, (py)C(R)NOH behave as *N*,*N* *'*-chelating ligands (see Scheme 2) making necessary the employment of additional inorganic or organic anions to complete the coordination sphere of the metal centre or to balance the charge of the complex cation. A variety of *mono*anions have been used for this reason, for example, PhCO_2_^−^ [21], Cl^−^ [22, 23], Br^−^ [24], and NO_3_^−^ [25]. Recently, we have started a research program to explore the use of the sulfate ion, SO_4_^2−^, in 3d-metal/2-pyridyl oxime chemistry, instead of the abovementioned *mono*anionic ligands. The possible advantages of using SO_4_^2−^ include (i) the possibility of triggering aggregation of preformed smaller species into new products, and (ii) the possible diversion of known reaction systems developed using inorganic *mono*anions to new species as a result of the higher charge and higher denticity of the sulfate ligand.

**Scheme 3:**
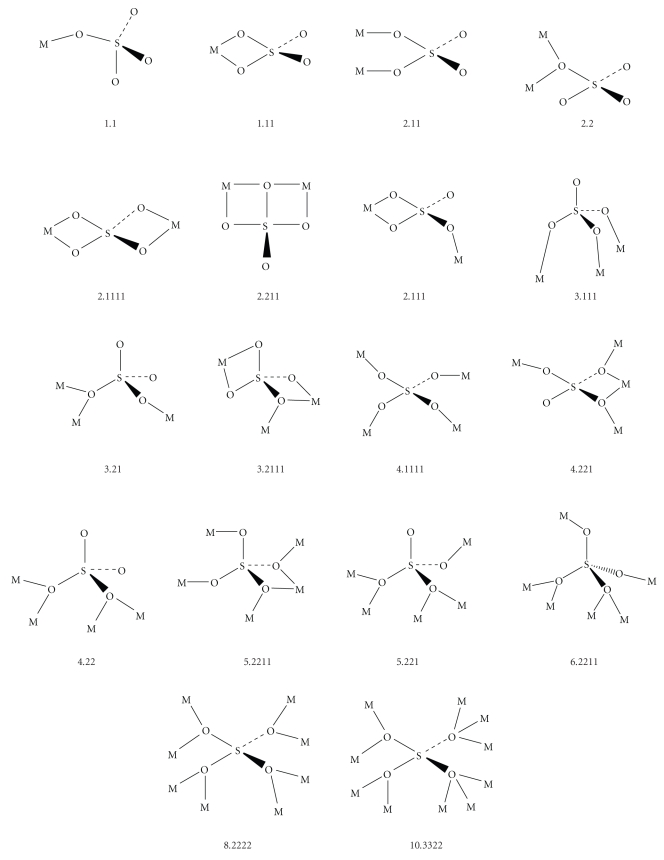
The up to now crystallographically established coordination modes of the sulfato ligand and the Harris notation [26] which describes these modes.

The sulfate ion [27] is currently a ligand of intense interest. The *μ*_2_, *μ*_3_, *μ*_4_, *μ*_5_, *μ*_8_,  or *μ*_10_ potential of SO_4_^2−^ (Scheme 3) prompted as to combine 2-pyridyloximes with the sulfate ligand to aim at new types of compounds.

In this paper, we report the synthesis and the X-ray structural characterization of the two new Mn(II) complexes [Mn_2_(SO_4_)_ 2 _{(py)C(Me)NOH}_4_]·(py)C(Me)NOH (**1**·(py)C(Me)NOH) and [Mn(HCO_2_)_2 _{(py)C(Me)NOH}_2_] (**2**) which contain the neutral methyl(2-pyridyl)ketone oxime as organic ligand. The IR data are discussed in terms of the nature of bonding and the structures of the two complexes.

## 2. Experiments

All manipulations were performed under aerobic conditions using materials and solvents as received. IR spectra were recorded on a Perkin-Elmer PC16 FT-IR spectrometer with samples prepared as KBr pellets.

**[Mn_2 _(SO_4_)_2 _{(py)C(Me)NOH}_4 _]·(py)C(Me)NOH (1·(py)C(Me)NOH)**

Solid MnSO_4_ · H_2_O (0.067 g, 0.40 mmol) was added to a slurry of (py)C(Me)NOH (0.054 g, 0.40 mmol) in H_2_O (15 cm^3^); the solid soon dissolved and the solution was stirred for 1 hour at room temperature. The resultant solution was left for slow evaporation. After one week, yellow crystals appeared which were collected by filtration, washed with cold H_2_O (1 cm^3^), cold MeOH (1 cm^3^) and ice-cold Et_2_O (2 cm^3^), and dried in air. The yield was 79% (based on the metal). Found %: C, 42.94; H, 3.89; N, 14.51. Calc % for C_35_H_40_O_13_N_10_S_2_Mn_2_: C, 42.78; H, 4.10; and N, 14.25. Selected IR data (KBr, cm^−1^): 3420 (wb), 3150 (m), 3069 (m), 2843 (m), 2363 (w), 2343 (w), 1654 (w), 1593 (s), 1561 (m), 1476 (s), 1437 (m), 1327 (m), 1285 (w), 1215 (m), 1124 (s), 1080 (s), 1030 (s), 1010 (s), 989 (s), 781 (s), 748 (m), 683 (m), 631 (m), 592 (m), 561 (w), 494 (w), 452 (w), and 447 (w).

**[Mn(HCO_2_ )_2_ {(py)C(Me)NOH}_2_ ](2)**

Solid NaOMe (0.090 g, 1.50 mmol) was added to a colourless solution of (py)C(Me)NOH (0.204 g, 1.50 mmol) in CH_2_Cl_2_ (20 cm^3^); the solid soon dissolved. Solid MnSO_4_·H_2_O (0.250 g, 1.50 mmol) was then added and the resulting solution was stirred for 24 hours at room temperature. A small quantity of undissolved material was removed by filtration and the dark brown filtrate layered with Et_2_O (40 cm^3^). Slow mixing gave X-ray quality yellow crystals of the product. The crystals were collected by filtration, washed with cold H_2_O (1 cm^3^), cold MeOH (2 cm^3^), and ice-cold Et_2_O (2 × 3 cm^3^), and dried in air. The yield was 45% (based on the metal). Found %: C, 46.95; H, 4.26; N, 13.43. Calc % for C_16_H_18_O_6_N_4_Mn: C, 46.82; H, 4.13; N, 13.98. Selected IR data (KBr, cm^−1^): 3412 (mb), 3073 (w), 2362 (m), 1846 (m), 1597 (s), 1562 (s), 1475 (s), 1436 (m), 1365 (s), 1348 (s), 1326 (m), 1250 (w), 1165 (w), 1137 (m), 1042 (s), 961 (m), 782 (s), 751 (s), 683 (m), 562 (w), and 458 (w).

### 2.1. X-Ray Crystallography

For **1**·(py)C(Me)NOH, X-Ray data were collected at 298 K using a Crystal Logic Dual Goniometer diffractometer with graphite-monochromated Mo-*K*_*a*_ radiation (*λ* = 0.71073 Å). Lorentz, polarization, and Ψ-scan absorption corrections were applied using Crystal Logic software. Symmetry equivalent data were averaged with *R*_int _ = 0.0084, to give 3727 independent reflections from a total 3964 collected. The structure was solved by direct methods and refined by full-matrix least-squares on F^2^, using 3727 reflections and refining 325 parameters. All nonhydrogen atoms were refined anisotropically. Hydrogen atoms were either located by difference maps and were refined isotropically or were introduced at calculated positions as riding on bonded atoms.

For **2**, X-ray data were collected at 100 K using a Oxford Diffraction diffractometer with graphite-monochromated Mo-*K*_*a*_ radiation (*λ* = 0.71073 Å). Symmetry equivalent data were averaged with *R*_int _ = 0.0160, to give 9343 independent reflections from a total of 13039 collected. The structure was solved by direct methods and refined by full-matrix least-squares on F^2^, using 9343 reflections and refining 258 parameters. All non-hydrogen atoms were refined anisotropically. Hydrogen atoms were either located by difference maps and were refined isotropically or were introduced at calculated positions as riding on bonded atoms.

Details of the data collection and refinement for **1**·(py)C(Me)NOH and **2** are given in Table 1.

## 3. Results and Discussion

### 3.1. Synthetic Comments

Treatment of MnSO_4_·H_2_O with one equivalent of (py)C(Me)NOH in H_2_O gave a colorless solution from which the new dinuclear compound [Mn_2 _(SO_4 _)_2 _{(py)C(Me)NOH}_4 _]·(py)C(Me)NOH (**1**·(py)C(Me)NOH) was obtained in ~80% yield. Its formation can be summarized in (1).

(1) $$\begin{array}{cc} & 2{\text{MnSO}}_{4}\cdot {\text{H}}_{2}\text{O}+5\left(\text{py}\right)\text{C}\left(\text{Me}\right)\text{NOH}\\ & \;\;\overset{{\text{H}}_{2}\text{O}}{\to }\left[{\text{Mn}}_{2}{\left({\text{SO}}_{4}\right)}_{2}{\left\{\left(\text{py}\right)\text{C}\left(\text{Me}\right)\text{NOH}\right\}}_{4}\right]\\ & \;\;\;\;\cdot \left(\text{py}\right)\text{C}\left(\text{Me}\right)\text{NOH}+2{\text{H}}_{2}\text{O}\\ & \;\;\;\;1\cdot \left(\text{py}\right)\text{C}\left(\text{Me}\right)\text{NOH}. \end{array}$$

The nonstoichiometric MnSO_4_·H_2_O to (py)C(Me)NOH reaction ratio (1 : 1) employed for the preparation of **1**·(py)C(Me)NOH (Section 2) did not prove detrimental to the formation of the complex. With the identity of **1**·(py)C(Me)NOH established by single-crystal X-ray crystallography, the “correct” stoichiometry (1 : 2.5) was employed and led to the pure compound in high yield.

As a next step, we decided to add base in the reaction mixture targeting the deprotonation of the organic ligand. Thus, treatment of MnSO_4_·H_2_O with one equivalent of (py)C(Me)NOH and one equivalent of NaOMe in CH_2_Cl_2_ gave a dark brown solution from which the mononuclear compound [Mn(HCO_2_)_2_{(py)C(Me)NOH}_2_] (**2**) was obtained. Its formation can be summarized in(2)

(2) $$\begin{array}{cc} & {\text{MnSO}}_{4}\cdot {\text{H}}_{2}\text{O}+2\left(\text{py}\right)\text{C}\left(\text{Me}\right)\text{NOH}+2\text{NaOMe}+2{\text{O}}_{2}\\ & \;\;\overset{{\text{CH}}_{2}{\text{Cl}}_{2}}{\to }\left[\text{Mn}{\left({\text{HCO}}_{2}\right)}_{2}{\left\{\left(\text{py}\right)\text{C}\left(\text{Me}\right)\text{NOH}\right\}}_{2}\right]\\ & \;\;+{\text{Na}}_{2}{\text{SO}}_{4}+3{\text{H}}_{2}\text{O}. \end{array}$$

To our surprise, an amount of the methoxide ions did not act as proton acceptors but they got oxidized to formates (HCO_2_^−^) during the aerial aggregation process [28]. Thus, the organic ligand in **2** is neutral. As expected, the nature of the base is crucial for the identity of the product; employment of NEt_3_, NMe_4_OH, NEt_4_OH, LiOH·H_2_O etc. leads to dark brown oily materials that have not been characterized. Also, note that: (i) The color of **2** (yellow) is different than the color of the reaction mixture (dark brown, this colour is characteristic of Mn^III^ or Mn^II/III^ species), and (ii) a similar reaction, but with (py)C(ph)NOH instead of (py)C(Me)NOH, yields the octanuclear mixed-valent cluster [Mn^II^_4_ Mn^III^_4_O_4_(NO_3_)_2_{(py)C(ph)NO}_8_(HCOO)_2_ (MeOH)_2_] [29] whose core consists of two butterfly subunits. These observations indicate that compound **2** is not the only product of the reaction and that, presumably, a higher nuclearity cluster, with the metals at higher oxidation states, is present in solution. Work is in progress to isolate the second product from the reaction mixture.

### 3.2. Description of Structures

**Figure 1:**
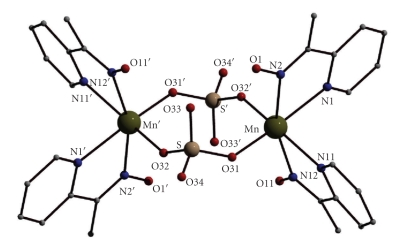
The dinuclear molecule present in **1**·(py)C(Me)NOH. Primes are used for the symmetry-related atoms.

**Figure 2:**
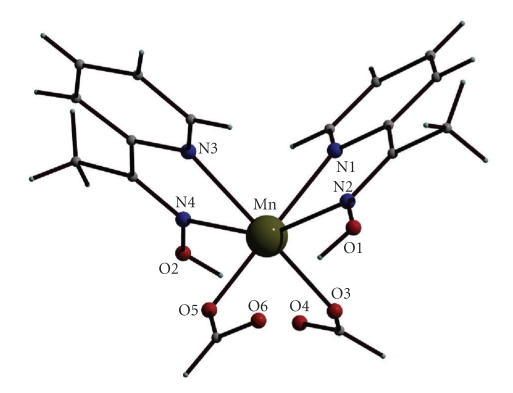
The molecular structure of compound **2**.

Selected interatomic distances and angles for complexes **1**·(py)C(Me)NOH and **2 **are listed in Tables 2 and 4, respectively. The molecular structures of the two compounds are shown in Figures 1 and 2.

Complex **1**·(py)C(Me)NOH crystallizes in the triclinic space group *P*-1. Its structure consists of dinuclear [Mn_2_(SO_4_)_2_{(py)C(Me)NOH}_4_] molecules and (py)C(Me)NOH molecules in the crystal lattice. The dinuclear molecules lie on a crystallographic inversion center. The two Mn^II^ atoms are bridged by two *η* ^1^: *η* ^1^: *μ*_2_ or 2.1100 (Harris notation [26]) sulfato ligands; two *N,N *′-chelating (py)C(Me)NOH ligands complete six coordination at each metal center. The ligating atoms of (py)C(Me)NOH are the nitrogen atoms of the neutral oxime and 2-pyridyl groups. Thus, adopting the Harris notation, (py)C(Me)NOH behaves as an 1.011 ligand.

The coordination sphere of the Mn^II^ ion in **1**·(py)C(Me)NOH exhibits a slightly distorted octahedral geometry as a consequence of the relatively small bite angles of the chelating ligands [N1–Mn–N2=70.11(10), N11–Mn–N12=70.19(10)°]. Both sulfato oxygen atoms O(31) and O(32′) are *trans* to the pyridyl nitrogen atoms N(1) and N(11), respectively. Each metal center adopts the *cis-cis-trans* configuration considering the position of the coordinated SO_4_^2−^ oxygen, pyridyl nitrogen and oxime nitrogen atoms, respectively. The *cis* arrangement of the oxime groups seems unfavourable, probably due to the steric hindrance arising from the methyl group upon oxime coordination. The long Mn ⋯ Mn*'* distance [5.040(2) Å] is a consequence of the presence of the two *syn*, *anti* sulfato bridges.

The molecular structure of **1**·(py)C(Me)NOH is stabilized by intramolecular hydrogen bonds (Table 3). Each coordinated (py)C(Me)NOH oxime group is strongly hydrogen bonded to an uncoordinated O atom of the sulfato ligand (O33 or O33′). Thus, O33 (and its symmetry equivalent) participates in two hydrogen bonds.

Complex **2 **crystallizes in the monoclinic space group P2_1_/n and its structure consists of mononuclear [Mn(HCO_2_)_2_{(py)C(Me)NOH}_2_] molecules. Two bidentate chelating (py)C(Me)NOH molecules (1.011 [26], see Scheme 2) and two monodentate HCO_2_^−^ ions create six-coordination at the Mn^II^ ion. The coordination geometry of the metal ion is distorted octahedral. As **1**·(py)C(Me)NOH, complex **2** is the *cis-cis*-*trans* isomer considering the positions of the coordinated HCO_2_^−^ oxygen, pyridyl nitrogen and oxime nitrogen atoms, respectively.

Intramolecular hydrogen bonds are present in the structure of **2 **(Table 5). The oximic oxygen atom of each (py)C(Me)NOH ligand is very strongly intramolecularly hydrogen bonded to one uncoordinated formate oxygen atom. 

Complexes **1**·(py)C(Me)NOH and **2** join a small but growing family of structurally characterized metal complexes containing the neutral or anionic forms of methyl(2-pyridyl)ketone oxime as ligands. The 1.011 ligation mode is the exclusive one for the metal complexes containing the neutral ligand [22, 24, 30]. 

The structurally characterized *Mn* complexes of (py)C(Me)NOH and/or (py)C(Me)NO^−^ [14, 31–33] are collected in Table 6, together with the cores of the polynuclear complexes and the ligands' coordination modes for convenient comparison. Closer inspection of Table 6 reveals that compound **1** is the first member of this subfamily in which the Mn^II^ ions are linked by the SO_4_^2−^ ion. Complex **2** can be compared with the compound [Mn^II^(O_2_CPh)_2_{(py)C(Me)NOH}_2_] [33] which contains terminal PhCO_2_^−^ ions, instead of HCO_2_^−^ in **2**; the HCO_2_^−^ versus PhCO_2_^−^ change has little structural effect.

### 3.3. IR Spectra

Complexes **1** and **2** exhibit medium to strong intensity IR bands at ~3400 cm^−1^, assignable to *ν*(*Ο*H) vibrations of the (py)C(Me)NOH molecules. The broadness and relatively low frequency of these bands are both indicative of hydrogen bonding.

The *ν*(C=N)_oxime_ and *ν*(N–O)_oxime_ vibrations for the free ligand appear as medium intensity bands at 1566 and 1116 cm^−1^, respectively [34, 35]. The 1116 cm^−1^ band is shifted to a lower wavenumber in **1** and **2** (**1**, 1080; **2**, 1042 cm^−1^). This shift is attributed to the coordination of the neutral oxime nitrogen [22]. The strong band at 1124 cm^−1^ in the spectrum of **1**·(py)C(Me)NOH should also have a *ν*(N–O)_oxime_ character resulting from the presence of lattice (py)C(Me)NOH molecules in the structure. Several bonds appear in the 1655-1400 cm^−1^ region for both complexes; contribution from the *ν*(C=N)_oxime_ and *δ*(OH) modes (>1580 cm^−1^) are expected in this region, but overlap with the stretching vibrations of the aromatic rings and the carboxylate groups (for **2**) renders assignments and discussion of the coordination shifts difficult.

The in-plane deformation band of the 2-pyridyl ring of free (py)C(Me)NOH at 637 cm^−1^ shifts upwards in **1 (**683 cm^−1^) and **2** (683 cm^−1^), confirming the involvement of the ring-N atom in coordination [36]. The appearance of a medium intensity band at 631 cm^−1^ in the spectrum of **1**·(py)C(Me)NOH is indicative of the presence of lattice (py)C(Me)NOH molecules in this complex.

The IR spectrum of the free, that is, ionic, sulfate (the SO_4_^2−^ ion belongs to the *T*_d_ point group) consists of two bands at ~1105 and ~615 cm^−1^, assigned to the *ν*_3_(*F*_2_) stretching [*ν*_d_(SO)] and *ν*_4_(*F*_2_) bending [*δ*_d_ (OSO)] modes, respectively [27, 37, 38]. The *ν*_1_(*A*_1_) stretching [*ν*_*s*_(SO)] and *ν*_2_(*E*) bending [*δ*d(OSO)] modes are not IR-active (these are Raman-active). The coordination of SO_4_^2−^ to metal ions decreases the symmetry of the group and the *ν*_3_ and *ν*_4_ modes are split [27, 37, 38]. In the case, the SO_4_^2−^-site symmetry is lowered from *T*_d_ to *C*_3v_ (monodentate coordination), both *ν*_1_ and *ν*_2_ appear in the IR spectrum with weak to medium intensity, while *ν*_3_ and *ν*_4_ each splits into two bands in both IR and Raman spectra [37]. When the SO_4_^2−^-site symmetry is lowered from *T*_d_ to *C*_2v_ (bidentate chelating or bridging coordination), again *ν*_1_ and *ν*_2_ appear in the IR spectrum (*ν*_2_ splits into two Raman modes), while *ν*_3_ and *ν*_4_ each splits into three IR-active and Raman-active vibrations [37]. The crystallographically established symmetry of the sulfato groups in **1**·(py)C(Me)NOH is *C*_2v_. The bands at 1215, 1124 and 1080 (overlapping with the N–O_oxime_ stretch) cm^−1^ are attributed to the *ν*_3_ modes [37, 39], while the bands at 592, 631 and 683 cm^−1^ (the latter two overlapping with the in-plane 2-pyridyl deformations) are assigned to the *ν*_4_ modes [37, 38]. The band at 1010 cm^−1^ and the two weak features at 494 and 452 cm^−1^ can be assigned to the *ν*_1_ and *ν*_2_ modes, respectively. The appearance of two *ν*_2_ bands is consistent with a symmetry at the sulfato groups lower than *C*_2v_ [37, 39]. Thus, from the vibrational spectroscopy viewpoint, the sulfato ligands of **1**·(py)C(Me)NOH appear to have *C*_i_ symmetry (and not *C*_2v_ as deduced from their bidentate character). When the SO_4_^2−^-site symmetry is lowered from *T*_d_ to *C*_i_, *ν*_3_ and *ν*_4_ each splits into three IR-active vibrations, *ν*_2_ splits into two ones, while *ν*_1_ appears as a single band [37, 39]. This spectroscopic feature in **1**·(py)C(Me)NOH is attributed to the fact that one uncoordinated oxygen atom of each bidentate bridging sulfate is hydrogen bonded to the oxygen atoms of the neutral oxime groups (see Table 3) resulting in a further lowering of the sulphate symmetry [39]. 

The *ν*(CO_2_) bands are difficult to assign in the spectrum of **2** due to the appearance of various stretching vibrations in the 1600–1400 cm^−1^ region and thus the application of the spectroscopic criterion of Deacon and Phillips [40] is very difficult.

## 4. Conclusions

The use of the sulfate ligand in combination with *neutral* (py)C(Me)NOH in Mn(II) chemistry has provided access to the two new neutral complexes [Mn_2 _(SO_4_)_2 _{(py)C(Me)NOH}_4 _]·(py)C(Me)NOH (**1**·(py)C(Me)NOH) and [Mn(HCO_2_)_2_{(py)C(Me)NOH}_2_] (**2**), the latter being sulphate-free. In both complexes, the organic ligand chelates through its nitrogen atoms. The sulfate anion bridges the two Mn^II^ atoms in **1**. Compounds 1 · (py)C(Me)NOH and **2** join a small family of structurally characterized manganese complexes containing the neutral or anionic forms of methyl(2-pyridyl)ketone oxime as ligands, while they are new examples of structurally characterized compounds in which (py)C(Me)NOH exists exclusively in its neutral form.

Analogues of **1**·(py)C(Me)NOH and **2** with phenyl(2-pyridyl)ketone oxime, (py)C(ph)NOH, are not known to date, and it is currently not evident whether the stability of these species is dependent on the particular nature of the 2-pyridyl oxime ligand. We are studying this matter. Synthetic efforts are also in progress to “activate” the *μ*_3_ to *μ*_6_ bridging potential of the sulfate ligand in Mn complexes containing 2-pyridyl oximes and/or their anions as a means to get access to clusters and polymers with interesting structural and magnetic properties. Studies on the biological activity of **1**·(py)C(Me)NOH and **2** are also planned.

## 5. Supplementary Information

CCDC 757892 and 757893 contain the supplementary crystallographic data for **1**·(py)C(Me)NOH and **2**. These data can be obtained free of charge via http://www.ccdc.cam.ac.uk/conts/retrieving.html, or from the Cambridge Crystallographic Data Centre, 12 Union Road, Cambridge CB2 1EZ, UK; fax: (+44)1223-336033; or e-mail: deposit@ccdc.cam.ac.uk.

## Figures and Tables

**Table 1:** Crystal data and structure refinement for **1**· (py)C(Me)NOH and 2.

Empirical formula	C_35_H_40_Mn_2_N_10_O_13_S_2_	C_16_H_18_MnN_4_O_6_
Formula weight	982.77	417.28
Crystal size	0.75 × 0.50 × 0.40	0.25 × 0.20 × 0.20
Crystal system	triclinic	monoclinic
Space group	P-1	P2_1_/n
**θ** range for data collection.°	5.5 ≤ *θ* ≤ 11.0	3.4 ≤ *θ* ≤ 30.1
*a*, Å	9.627(4)	10.6538(5)
*b*, Å	9.962(4)	14.3935(7)
*c*, Å	11.750(4)	11.8231(8)
*α*,°	92.610(10)	90.00
*β*, °	96.560(10)	90.264(7)
*γ*, °	107.450(10)	90.00
*V*, Å^3^	1064.2(7)	1813.00(17)
*Z*	1	4
*ρ*_calcd_, gcm^−3^	1.534	1.529
*μ*, mm^−1^	0.766	0.770
*GOF*	1.116	1.009
*R*1^a^	0.0443	0.0344
*w* *R*2^b^	0.1125	0.0948

^a^ *I* > 2*σ*(*I*), *R*_1_ = ∑(|*F*_o_ | −|*F*_c_|)/∑(|*F*_o_|)

^b^ *w* *R*_2_ = {∑[*w*(*F*_o_^2^−*F*_c_^2^)^2^]/∑[*w*(*F*_o_^2^)^2^]}^1/2^

**Table 2:** Selected dond lengths (Å) and angles (°) for **1**·(py)C(Me) *N* *O* *H*.^*a*^

Mn–O31	2.089(2)	Mn–N2	2.287(3)
Mn–O32′	2.102(3)	Mn–N11	2.300(3)
Mn–N1	2.287(3)	Mn–N12	2.283(3)
O31–Mn–O32′	101.06(11)	O32′–Mn–N12	94.84(11)
O31–Mn–N1	164.21(11)	N1–Mn–N2	70.11(10)
O31–Mn–N2	95.50(11)	N1–Mn–N11	88.37(10)
O31– Mn–N11	87.21(10)	N1–Mn–N12	94.52(10)
O31–Mn–N12	98.23(10)	N2–Mn–N11	97.89(10)
O32′–Mn–N1	87.06(11)	N2–Mn–N12	161.24(10)
O32′–Mn–N2	95.00(11)	N11–Mn–N12	70.19(10)
O32′–Mn–N11	163.96(11)		

^a^Primes denote symmetry-related atoms.

**Table 3:** Dimensions of the hydrogen bonds in complex **1**·(py)C(Me) *N* *O* *H*.^*a*^

D–H⋯A	D⋯A	H⋯A	D–H⋯A	Symmetry Operator of A
	[Å]	[Å]	[°]	

O(1)–H(O1)⋯O(33)	2.644	1.967	154.5	*x*, *y*, *z*
O(11′)–H(O11′ )⋯O(33)	2.598	1.720	174.8	*x*, *y*, *z*

^**a**^ **A**= acceptor, D = donor.

**Table 4:** Selected dond lengths (Å) and angles (°) for 2.

Mn–O3	2.125(1)	Mn–N2	2.248(1)
Mn–O5	2.091(1)	Mn–N3	2.305(1)
Mn–N1	2.272(1)	Mn–N4	2.264(1)
O3–Mn–O5	95.48(5)	O5–Mn–N4	95.61(4)
O3–Mn–N1	87.76(5)	N1–Mn–N2	70.98(4)
O3–Mn–N2	94.27(5)	N1–Mn–N3	89.65(5)
O3–Mn–N3	172.60(5)	N1–Mn–N4	90.78(5)
O3–Mn–N4	102.46(5)	N2–Mn–N3	91.43(5)
O5–Mn–N1	172.03(5)	N2–Mn–N4	154.80(5)
O5–Mn–N2	101.47(5)	N3–Mn–N4	70.64(5)
O5–Mn–N3	87.99(4)		

**Table 5:** Dimensions of the hydrogen bonds in complex 2.^*a*^

D–H⋯A	D⋯A	H⋯A	D–H⋯A	Symmetry Operator of A
	[Å]	[Å]	[°]	

O(1)–H(O1)⋯O(6)	2.542	1.710	167.2	*x*, *y*, *z*
O(2)–H(O2)⋯O(4)	2.585	1.777	165.4	*x*, *y*, *z*

^a^A = acceptor, D = donor.

**Table 6:** Formulae, coordination modes of the ligands, and cores of the structurally characterized Mn complexes of (py)C(Me)NOH and/or (py)C(Me)NO^−^.

Complex^a^	Coordination modes^b^	Core^c^	Ref.
[Mn^III^_3_O(O_2_CMe)_3_{(py)C(Me)NO}_3_]^+^	2.111	[Mn_3_(*μ*_3_-O)]^7+^	[14]
[Mn^III^_3_O(O_2_CEt)_3_{(py)C(Me)NO}_3_]^+^	2.111	[Mn_3_(*μ*_3_-O)]^7+^	[14]
[Mn^III^Cl_2_{(py)C(Me)NO}{(py)C(Me)NOH}_2_]	1.011		[32]
[Mn^II^(O_2_CPh)_2_{(py)C(Me)NOH}_2_]	1.011		[33]
[Mn^II^_4_Mn^III^_4_O_2_(OH)_2_(O_2_CPh)_10_{(py)C(Me)NO}_4_]	2.111	[Mn_8_(*μ*_4_-O)_2_(*μ*_3_-OH)_2_]^14+^	[33]
[Mn^II^_2_Mn^IV^(OMe)_2_{(py)C(Me)NO}_4_Br_2_]	2.111	[Mn_3_(*μ*_3_-OMe)_2_]^6+^	[31]
[Mn^II^_2_Mn^III^_6_O_4_(OMe){(py)C(Me)NO}_9_{(py)C(Me)NOH}]^4+^	2.111, 3.211	[Mn_8_(*μ*_3_-O)_4_(*μ*-OMe)(*μ*-OR*'* *'*)]^11+d^	[31]
[Mn^II^_2_(SO_4_)_2_{(py)C(Me)NOH}_4_]	1.011		This work
[Mn^II^(HCO_2_)_2_{(py)C(Me)NOH}_2_]	1.011		This work

^a^Counterions and lattice solvent molecules have been omitted; ^b^ using the Harris notation [26]; ^c^only for the polynuclear complexes; ^d^ R′′ = (py)C(Me)N.
